# Association of Serum Sodium Levels and Delirium in Patients with Sepsis: A Retrospective Study

**DOI:** 10.3390/biomedicines14020410

**Published:** 2026-02-11

**Authors:** Meiying Wang, Xi Wu, Mengqi Du, Dongjun Tie, Younian Xu

**Affiliations:** 1 Department of Anesthesiology, Union Hospital, Tongji Medical College, Huazhong University of Science and Technology, Wuhan 430022, China; docwangmy@163.com (M.W.); wu2018whuh@163.com (X.W.); mengqidu1326@163.com (M.D.); 15008616505@163.com (D.T.); 2 Institute of Anesthesia and Critical Care Medicine, Union Hospital, Tongji Medical College, Huazhong University of Science and Technology, Wuhan 430022, China; 3 Key Laboratory of Anesthesiology and Resuscitation, Huazhong University of Science and Technology, Ministry of Education, Wuhan 430022, China

**Keywords:** sepsis, delirium, sodium levels, MIMIC-IV database, hypernatremia, hyponatremia

## Abstract

**Background**: This study aims to elucidate the relationship between serum sodium and the risk of sepsis-associated delirium (SAD), with particular emphasis on the critical threshold of 138.4 mmol/L. **Methods**: The retrospective study utilized data from the MIMIC-IV database. The analysis focused on serum sodium concentrations measured within the first 24 h of ICU admission. The association between sodium levels and the risk of delirium was assessed using restricted cubic spline (RCS) analysis and multivariable logistic regression. Subgroup analyses and propensity score matching (PSM) were used to mitigate potential confounding factors. **Results**: A total of 7356 septic patients were included, with 1861 (25.3%) developing delirium. RCS analysis revealed a significant non-linear relationship between sodium levels and delirium risk, with a threshold at 138.4 mmol/L. Sodium levels ≤ 138.4 mmol/L were associated with a reduced risk of delirium (OR 0.97, 95% CI: 0.95–0.99, *p* = 0.041), while levels > 138.4 mmol/L significantly increased the risk of delirium (OR 1.08, 95% CI: 1.06–1.11, *p* < 0.001). After PSM, hypernatremia was associated with a higher delirium incidence than hyponatremia (55.00% vs. 36.67%, *p* = 0.044) and remained an independent risk factor for delirium in logistic regression (OR 2.89, 95% CI 1.17–7.18, *p* = 0.022). **Conclusions**: This study identified a non-linear, threshold-dependent association between serum sodium and delirium susceptibility in septic patients, with 138.4 mmol/L as a critical tipping point. Hypernatremia emerged as a more potent risk factor for delirium compared to hyponatremia. These findings underscore the importance of sodium management in septic patients and suggest that serum sodium may serve as a potential biomarker for predicting neuropsychiatric outcomes in sepsis.

## 1. Introduction

Sepsis is a critical systemic response to infection and a major cause of high mortality and morbidity in intensive care units (ICUs) worldwide [1]. Despite significant advancements in sepsis research, the pathogenesis of sepsis remains incompletely understood, and current treatment options are largely limited to antibiotic therapy, fluid resuscitation, and organ support [2]. Sepsis affects over 30 million individuals annually, leading to longer hospital stays, increased economic burden, and remains a significant global health concern [3]. Multiple organ failure is a key characteristic and the leading cause of mortality in sepsis [4]. Brain dysfunction, a severe complication of sepsis and part of multiple organ failure, resulting from intricate pathophysiological mechanisms, such as neuroinflammation, hypoxemia, blood–brain barrier (BBB) disruption, and metabolic dysregulation [5]. Clinically, delirium is the most common clinical manifestation in sepsis patients, with an incidence rate as high as 70%, and is referred to as sepsis-associated delirium (SAD) [6,7]. Compared with postoperative delirium (4–53%), the markedly higher incidence of SAD highlighting the severe clinical burden of SAD [8]. SAD is characterized by disturbances in consciousness and attention, cognitive deficits, emotional lability, abnormal behavior, and sleep–wake cycle disruption [9]. Furthermore, SAD is associated with increased mortality, irreversible brain damage, prolonged hospitalization and higher healthcare costs in sepsis patients [10]. Recently, the Society of Critical Care Medicine (SCCM) has recommended using delirium assessment tools such as the Confusion Assessment Method for the ICU (CAM-ICU) or the Intensive Care Delirium Screening Checklist (ICDSC) for diagnosing delirium in the ICU [11]. However, early biological biomarkers for identifying high-risk factors for SAD remain limited [12]. Consequently, further exploring the risk factors and the biological indicators of delirium in sepsis patients remains a clinical challenge.

Sodium, a pivotal electrolyte in extracellular fluid, plays a crucial role in maintaining extracellular osmotic pressure and transmembrane potential [13]. Serum sodium levels (SLs) are tightly regulated in humans; however, imbalances under pathological conditions can increase the risk of dysnatremia [14]. Rapid fluctuations in serum sodium levels can cause acute neurological damage, manifested as seizures, headaches, and disturbances in consciousness [15]. Dysnatremia in acute stroke and traumatic brain injury is associated with a poor prognosis [16,17]. Moreover, emerging evidence has demonstrated associations between hypernatremia, hyponatremia, and postoperative delirium (POD) [18,19]. Collectively, these findings indicate a potential link between serum sodium levels and brain dysfunction. However, despite the high morbidity and poor prognosis with SAD in sepsis patients, few studies have specifically explored the relationship between serum sodium levels and sepsis-associated delirium (SAD).

In this context, the present retrospective study aimed to evaluate the potential correlation between serum sodium levels and clinical outcomes in septic patients with SAD. This investigation sought to provide new insights into delirium biomarkers and prognosis, ultimately elucidating the significance of human serum sodium levels in sepsis patients.

## 2. Methods

### 2.1. Data Selection

Clinical data in this study were sourced from the MIMIC-IV database (version 3.1), which encompasses detailed health information for over 190,000 patients admitted to the Beth Israel Deaconess Medical Center’s ICU between 2008 and 2022. This comprehensive dataset facilitates large-scale investigations of ICU patient populations [20]. One of the authors (WMY) completed the requisite online training course provided by the National Institutes of Health (NIH) and was granted access to the database (No. 69192968); further details are provided in Additional File S1. All personal information within the MIMIC database has been de-identified, and ethical approval for its use was obtained from the Institutional Review Board of Beth Israel Deaconess Medical Center (No. 2001P-001699/14). Given that no additional personal data were collected in the present study, further ethical approval and written informed consent were deemed unnecessary. A total of 30,634 patients with sepsis were identified from the MIMIC-IV database according to the Sepsis-3 criteria. After applying the exclusion criteria, 22,444 patients were removed for the following reasons: younger than 18 years of age (n = 0), non-first ICU admission (n = 1475), ICU stay of less than 24 h (n = 1596), or missing serum sodium measurements (n = 19,373). After these exclusions, 8190 patients remained eligible for further evaluation. Additional screening excluded 833 patients, either due to a documented diagnosis of dementia (n = 175) or treatment with continuous renal replacement therapy (CRRT) (n = 658). Ultimately, 7356 patients satisfied all inclusion criteria and were included in the final cohort. These individuals were subsequently divided into two groups based on the presence or absence of delirium: 1861 in the delirium cohort and 5495 in the non-delirium cohort.

### 2.2. Data Collection

Data extraction was conducted from the MIMIC-IV database using Structured Query Language (SQL) in PostgreSQL (version 14.2). The extracted data represented baseline characteristics of patients during the first 24 h of ICU admission. Demographic characteristics included age, sex, race, and body weight. Comorbidities documented included acute kidney injury (AKI), myocardial infarction, congestive heart failure, cerebrovascular disease, chronic pulmonary disease, diabetes, renal disease, liver disease, and malignant cancer were documented. Laboratory parameters included glucose, lactate, blood urea nitrogen (BUN), creatinine, bicarbonate, chloride, calcium, potassium, white blood cell (WBC) count, platelet count, red blood cell (RBC) count, urine output, and sodium levels (first recorded, minimum, maximum, and average values). Unless otherwise specified, laboratory variables were defined as the first available measurement within the first 24 h of ICU admission. Disease severity at admission was assessed using the Simplified Acute Physiology Score II (SAPS II), the Charlson Comorbidity Index, and the Sequential Organ Failure Assessment (SOFA) score. Outcomes evaluated included hospital length of stay (Hospital-LOS), ICU length of stay (ICU-LOS), hospital mortality, ICU mortality, and 28-day mortality.

Missing data were imputed using the missForest (version 1.6.1) R package, which effectively handles mixed-type ICU variables and captures non-linear relationships. Variables with a missing rate exceeding 15% (e.g., ALT, AST, BMI, C-reactive protein, neutrophil count, and lymphocyte count) were excluded from further analysis. For variables with missing rates below 15% (e.g., WBC, RBC, platelet count, BUN, creatinine, bicarbonate, chloride, calcium, potassium) imputation was performed to minimize potential bias.

### 2.3. Exposure and Outcome

The primary exposure was defined as the first serum sodium measurement within 24 h of ICU admission, selected to capture early sepsis-related pathophysiology and to minimize confounding from subsequent ICU interventions.

The primary outcome was the occurrence of SAD during the patient’s ICU admission. Secondary outcomes included hospital length of stay (Hospital-LOS), ICU length of stay (ICU-LOS), hospital mortality, ICU mortality, and 28-day mortality. To ensure diagnostic accuracy, delirium assessment was restricted to patients with a Richmond Agitation and Sedation Scale (RASS) score ≥ −3; those with scores < −3, indicative of deep coma, were excluded. Delirium was defined as the occurrence of at least one positive CAM-ICU assessment during ICU admission, with all available CAM-ICU records utilized to determine the outcome [21].

### 2.4. Statistical Analyses

In the baseline analysis, patients were initially classified based on the occurrence of delirium. Further stratification was conducted, according to serum sodium levels, dividing patients into three groups: hyponatremia (<135 mmol/L), normal (135–145 mmol/L), and hypernatremia (>145 mmol/L). Continuous variables were presented as means ± standard deviations or medians with interquartile ranges, contingent upon depending on data distribution, while presented categorical variables were presented as counts with percentages. Comparisons between groups were performed using independent samples *t*-tests or Mann–Whitney U tests for continuous variables, and chi-square or Fisher’s exact tests for categorical variables.

To explore the potential non-linear association between sodium levels and the risk of delirium, restricted cubic spline (RCS) curves were used. Four standard knots were placed at the 0.05, 0.35, 0.65, and 0.95 percentiles of the sodium level distribution. The spline-derived threshold was determined via two-piecewise logistic regression in R, with the breakpoint selected by maximizing the log-likelihood and minimizing the Akaike information criterion (AIC). Subsequently, stratified logistic regression analyses were conducted for sodium levels both below and above the identified threshold.

To examine the association between sodium disturbances and delirium risk, we developed multivariable logistic regression models. Model 1 served as the unadjusted baseline. Model 2 was adjusted for demographic characteristics and comorbidities. Model 3 further incorporated laboratory indices. Model 4 additionally accounted for midazolam administration, mechanical ventilation and SOFA score. Odds ratios (ORs) and 95% confidence intervals (CIs) were reported for all models, all subsequent analyses refer to this section. To further mitigate confounding effects, we performed propensity score matching (PSM) to compare patients with hypernatremia versus hyponatremia. Propensity scores were estimated using a multivariable model encompassing demographic, clinical, and laboratory variables. Nearest-neighbor matching was conducted at a 1:1 ratio with a caliper width of 0.2 without replacement. Covariates balance after matching was evaluated using standardized mean differences. A conditional logistic regression model was subsequently applied to the matched cohort to re-assess the association between sodium status and the risk of delirium.

We further performed univariate and multivariate subgroup analyses to assess the robustness of our findings and explore potential interactive factors. Subgroups were stratified by age, sex, SOFA score and presence of comorbidities, including acute kidney injury, congestive heart failure, diabetes, renal disease, chronic pulmonary disease and liver disease. Additionally, ICU interventions, such as midazolam exposure and mechanical ventilation status were considered.

All statistical analyses were performed using Stata (version 15.0 SE) and R (version 4.2.2). A two-sided *p*-value < 0.05 was considered statistically significant.

## 3. Results

### 3.1. Study Population and Baseline Characteristics

**Figure 1:**
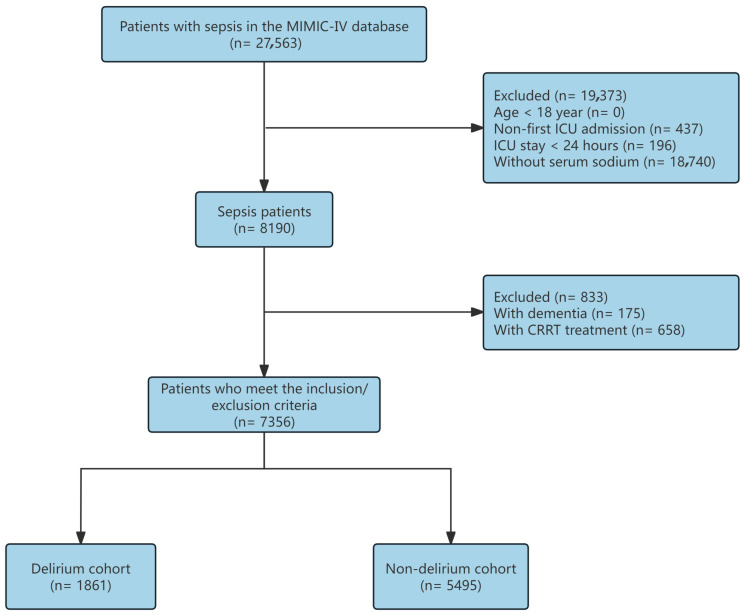
Study design flowchart.

Among 7356 eligible patients (Figure 1), delirium developed in 1861 (25.3%) during ICU stay. Baseline characteristics are summarized in Table 1. While age did not differ significantly between groups, male sex was more prevalent in the delirium cohort (38.7% vs. 33.4%). Patients with delirium presented with markedly greater illness severity and comorbidity burden, median [IQR] for each group (SOFA 4 [IQR 3–6] vs. 3 [2–5]; SAPS II 42 [34–52] vs. 37 [31–45]; Charlson index 5 [3–7] vs. 4 [2–6]). They also experienced higher rates of AKI (90.9% vs. 77.6%) and cerebrovascular disease (19.7% vs. 10.4%), more severe metabolic disturbances (elevated lactate and BUN/creatinine, decreased bicarbonate and urine output), and greater midazolam administration (27.2% vs. 11.7%). Notably, initial serum sodium was modestly reduced in the delirium group (136.1 ± 6.5 vs. 137.1 ± 4.2 mmol/L). Delirium correlated with substantially prolonged ICU and hospital stays and increased mortality across all measured timepoints.

### 3.2. Non-Linear Relationship Between Sodium Levels and the Risk of Delirium

**Figure 2:**
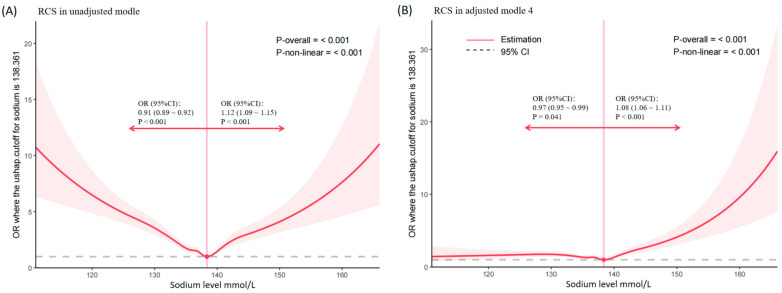
Non-linear Association Between Serum Sodium Levels and Delirium Risk. (**A**) illustrates the results of the unadjusted model, (**B**) presents the results fully adjusted results from Model 4. The red solid line denotes the odds ratio (OR), the pink dashed lines indicate the upper and lower bounds of the 95% confidence interval (95% CI), and the shaded pink area represents the confidence interval region. The horizontal dashed line represents the null value (OR = 1).

Figure 2 presents the restricted cubic spline (RCS) analysis results, illustrating the non-linear association between sodium levels and delirium risk in septic patients. The curve demonstrates a significant overall non-linear relationship (*p* for overall association ≤ 0.001, *p* for non-linear association ≤ 0.001). Specifically, sodium levels ≤ 138.4 mmol/L were associated with a reduced risk of delirium (OR 0.91; 95% CI: 0.89–0.92, *p* < 0.001), but sodium levels > 138.4 mmol/L were associated with an increased risk of delirium (OR 1.12; 95% CI: 1.09–1.15, *p* < 0.001). In the fully adjusted model (Model 4), sodium levels ≤ 138.4 mmol/L continued to demonstrate a protective effect (OR 0.97; 95% CI: 0.95–0.99, *p* = 0.041), and sodium levels > 138.4 mmol/L remained associated with elevated delirium risk (OR 1.08; 95% CI: 1.06–1.11, *p* < 0.001). Detailed logistic regression results are provided in Additional File S2 (Table S1).

### 3.3. Clinical Outcomes in Different Sodium Levels

Patients were categorized into three groups based on serum sodium levels: hyponatremia (<135 mmol/L), normal sodium (135–145 mmol/L), and hypernatremia (>145 mmol/L) groups. The incidence of delirium was highest in the hypernatremia group (53.85%), followed by the hyponatremia group (36.91%), and lowest in the normal sodium group (21.25%) (*p* < 0.001). Hospital and ICU length of stay (LOS) were longest in the hyponatremia group (11.11 days and 3.55 days, respectively), followed by the hypernatremia group (10.25 days and 4.19 days), and shortest in the normal sodium group (7.29 days and 2.31 days) (*p* < 0.001). Mortality rates, including hospital, ICU, and 28-day mortality, were highest in the hypernatremia group (38.46%, 30.77%, and 42.31%, respectively) (all *p* < 0.001). These findings are summarized in Table 2.

The findings from the multimodal logistic regression analysis are presented in Table 3. Our study revealed a significant association between both hypernatremia and hyponatremia with the development of delirium, and the impact of hypernatremia is more obvious than hyponatremia. After controlling for baseline comorbidities, laboratory indices, SOFA score, and ICU exposures (midazolam and mechanical ventilation), hypernatremia persisted as an independent risk factor for delirium (OR 3.37, 95% CI 2.28–5.0). Additionally, sodium concentrations were grouped into quartiles, with the lowest odds ratio (OR) observed in the Q3 group. Detailed results are presented in Additional File S2 (Table S1).

### 3.4. Propensity Score Matching

The PSM-matched cohort is presented in Table 4, baseline characteristics were well balanced between the hyponatremia and hypernatremia groups (all *p* > 0.05), with the exception of creatinine levels, which were higher in the hypernatremia group (*p* = 0.020). The incidence of delirium was significantly higher in the hypernatremia group compared to the hyponatremia group (55.00% vs. 36.67%, *p* = 0.044). However, no significant differences were observed in hospital mortality, ICU mortality, or 28-day mortality between the two groups (all *p* > 0.05). Patients in the hypernatremia group had a longer hospital length of stay (LOS) (15.46 days) compared to the hyponatremia group (11.91 days), but this difference was not statistically significant (*p* = 0.261). Similarly, ICU LOS was not significantly different between the two groups (*p* = 0.696).

**Figure 3:**
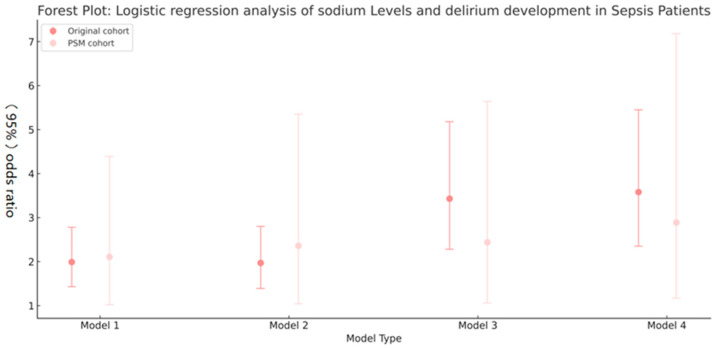
Logistic regression analysis of sodium levels and delirium risk in sepsis patients.

Logistic regression analysis of the PSM cohort revealed that hypernatremia was significantly associated with an increased risk of delirium relative to hyponatremia. In the fully adjusted model (Model 4), the odds ratio for delirium in the hypernatremia group was 2.89 (95% CI: 1.17–7.18, *p* = 0.022), compared with hyponatremia. These results are depicted in Figure 3.

The figure depicts the association between hypernatremia and the delirium risk, comparing the original cohort with the PSM-matched cohort across different model specifications (detailed in Table 3).

### 3.5. Subgroup Analysis

**Figure 4:**
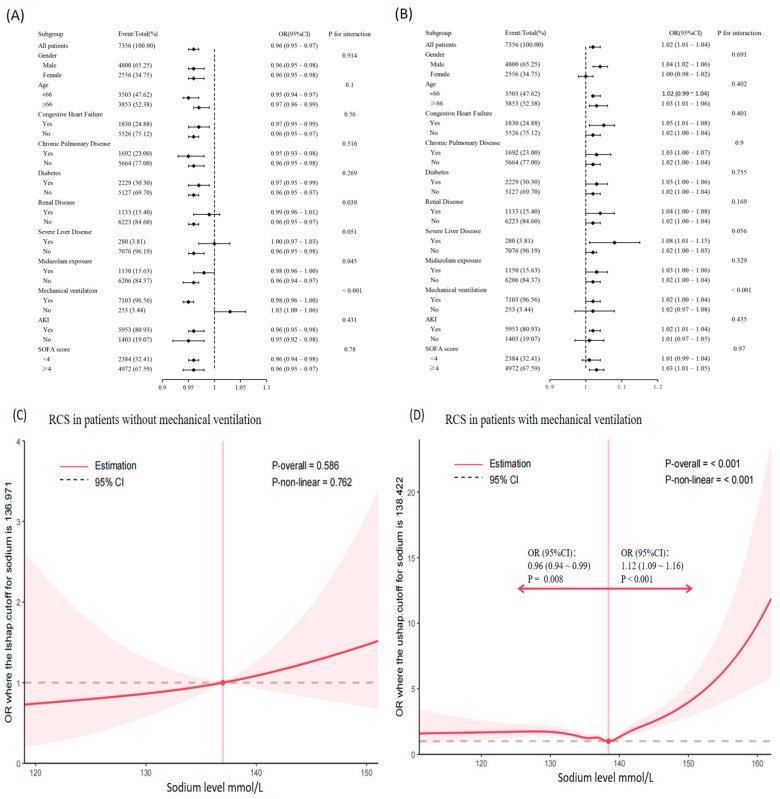
Subgroup analysis results. (**A**,**B**) Forest plots show the results of the subgroup analysis using the unadjusted model (**A**) and the fully adjusted model 4 (**B**) from Table 3. (**C**,**D**) The restricted cubic spline (RCS) curves illustrate the relationship between sodium levels and the risk of delirium in patients without mechanical ventilation (**C**) and those with mechanical ventilation (**D**).

In the unadjusted model, significant interactions were identified between sodium levels and renal disease (*p* = 0.039), pyramidal exposure (*p* = 0.045), and mechanical ventilation (*p* < 0.001). No other significant interactions were observed (all *p* > 0.05), as shown in Figure 4A. After adjusting for potential confounders, the association between mechanical ventilation and delirium remained significant (*p* < 0.001). However, the interactions with midazolam exposure (*p* = 0.329) and renal disease (*p* = 0.169) were no longer significant. The adjusted results are presented in Figure 4B.

To further explore the potential interaction between sodium levels and mechanical ventilation, the cohort was stratified into two groups: patients who received mechanical ventilation and those who did not. The restricted cubic spline (RCS) analysis in the non-mechanical ventilation group reveal no significant non-linear relationship between sodium levels and delirium (*p* for non-linearity > 0.05, Figure 4C). Conversely, in the mechanical ventilation group, a significantly non-linear relationship was observed, with a threshold identified at a sodium concentration of approximately 138.4 mmol/L (Figure 4D). Detailed logistic regression results are provided in Additional File S2.

## 4. Discussion

In this study, a significant nonlinear association was observed between serum sodium concentration and delirium risk in septic patients, with 138.4 mmol/L serving as a critical inflection point: concentrations below this threshold were protective, whereas higher levels conferred increased risk. Notably, hypernatremia emerged as a robust predictor of adverse outcomes. These findings highlight that sodium dysregulation, particularly hypernatremia, represents a crucial modifiable factor in the pathogenesis of sepsis-associated delirium.

The contribution of electrolyte abnormalities to cognitive dysfunction has garnered increasing attention in recent research [16,22]. Piccirillo et al. identified electrolyte disturbances, as components of systemic dysfunction, as important contributors to delirium development [23]. Sodium ions are essential for maintaining osmotic balance, transmembrane electrical gradients, and neuronal excitability [24,25]. Elevated extracellular sodium may potentiate inflammatory signaling through NLRP3 inflammasome activation and oxidative stress pathways, thereby amplifying the systemic inflammatory milieu of sepsis and exacerbating neuronal injury [26]. This positions electrolyte imbalance as an upstream risk factor for delirium. Complementing this mechanism, Schramm et al. demonstrated that impaired cerebral autoregulation (AR) and microvascular dysfunction constitute central pathophysiological mechanisms underlying SAD [27]. Sodium dysregulation has been shown to compromise cerebral vascular autoregulatory capacity [27], suggesting that hypernatremia may exacerbate SAD by disrupting cerebral hemodynamic stability and promoting microvascular dysfunction.

Consistent with our findings, a study by Romain Sonneville demonstrated that hypernatremia independently predicted delirium risk (OR = 2.30, 95% CI 1.48–3.57) [28]. Extending this observation, our analysis further revealed a nonlinear U-shaped relationship between serum sodium and delirium risk, with a critical threshold at 138.4 mmol/L. While Esra Uyar et al. reported that hyponatremia may induce cerebral edema and cognitive impairment, thereby increasing delirium susceptibility [29], our findings indicate that hypernatremia exhibits a stronger association with adverse outcomes in septic patients, particularly regarding SAD risk. Notably, hypernatremia was relatively uncommon in our cohort, which may limit the precision of mortality estimates for this subgroup; accordingly, these findings should be interpreted cautiously. Nowak et al. previously reported that both hyponatremia (<135 mmol/L) and hypernatremia (>145 mmol/L) were associated with cognitive decline in healthy older men [30]. By contrast, our RCS analysis identified an inflection point at 138.4 mmol. This value lies within the conventional normal range (135–145 mmol/L). This suggests that the neurological vulnerability to sodium fluctuations is context-dependent, with critical illness potentially lowering the threshold for sodium-induced neuronal injury. Clinically, these findings imply that clinicians should monitor sodium levels beyond overt dysnatremia, attending to upper-normal ranges in high-risk septic patients. Enhanced surveillance and meticulous regulation of water-electrolyte balance are warranted in this population. Nevertheless, serum sodium should be regarded as a marker of physiological stress rather than an isolated therapeutic target, and delirium prevention strategies should be grounded in comprehensive clinical assessment.

Univariate subgroup analysis revealed an interaction between midazolam use and serum sodium concentration, suggesting that sedative exposure may modulate cerebral vulnerability to sodium-related injury. This finding aligns with Zhou et al., who demonstrated that midazolam administration significantly increases delirium risk in critically ill patients [31]. Clinically, midazolam is preferentially administered to patients with greater disease severity, prolonged mechanical ventilation, or multiple organ dysfunction-all established independent risk factors for delirium [32]. After adjusting for these confounding clinical variables, the interaction between midazolam and serum sodium disappeared, indicating that the univariate association likely reflects underlying illness severity and treatment complexity rather than pharmacologic modulation of sodium-related brain vulnerability. In contrast, the interaction between mechanical ventilation and serum sodium persisted across both univariate and multivariate analyses. Although mechanical ventilation is a well-documented independent risk factor for delirium [33], stratified analyses revealed that the U-shaped relationship between serum sodium concentration and delirium risk remained robust among ventilated patients, with an identical threshold of 138.4 mmol/L. We speculate that this apparent interaction may be attributable to the limited sample size of non-ventilated patients, rather than genuine effect modification. Mechanically, ventilated patients typically exhibit more severe systemic inflammation and hemodynamic instability, which may impair microcirculatory perfusion and thereby reduce tolerance to osmotic stress [34]. Concomitantly, gas-exchange abnormalities and arterial CO_2_ fluctuations can alter cerebral blood flow, amplifying the neurological impact of dysnatremia [35]. Furthermore, ventilated patients more frequently receive analgesic-sedative agents and aggressive fluid–vasopressor resuscitation, which may precipitate rapid osmolar shifts and compromise blood–brain barrier integrity.

Our study has several limitations warranting consideration. First, the observational design precludes causal inference; although our findings demonstrated a robust association between dysnatremia and delirium, they do not establish that sodium correction prevents this outcome. Randomized controlled trials or well-designed prospective studies are needed to evaluate the therapeutic potential of sodium modulation. Second, with the MIMIC-IV database offers a large, granular cohort of critically ill patients, its single-center nature may limit generalizability to more diverse clinical populations and healthcare systems. Third, our analysis was restricted to the acute phase of sepsis, examining only the association between sodium levels and the incident delirium during ICU stay; we did not assess long-term cognitive trajectories or prognostic implications beyond hospital discharge. Finally, hypoactive delirium may be under-recognized during routine ICU assessments using the CAM-ICU, potentially resulting in misclassification bias, underestimation of its true incidence, and attenuation of observed effect estimates. Future studies incorporating comprehensive delirium phenotyping and continuous electrophysiological monitoring may help mitigate this detection bias.

## 5. Conclusions

Despite growing recognition of sepsis-associated delirium as a critical determinant of short- and long-term outcomes, the contribution of potentially modifiable systemic factors-particularly serum sodium-has remained incompletely characterized. In this study, we identified a non-linear relationship between serum sodium and delirium risk in septic patients, with a critical threshold identified at 138.4 mmol/L and hypernatremia demonstrating a stronger association than hyponatremia. These findings suggest that early serum sodium levels may serve as a readily available biomarker for delirium risk stratification in sepsis. However, whether targeted sodium modulation can prevent SAD remains to be established through prospective interventional studies.

## Figures and Tables

**Table 1:** Demographic and clinical characteristics of sepsis patients with and without delirium.

Variables	Overall	Non-Delirium	Delirium	*p*
Number	7356	5495	1861	
Age, years	65.79 ± 14.39	65.81 ± 13.98	65.74 ± 15.54	0.864
Gender, *n* (%)				<0.001
Male	2556 (34.75)	1835 (33.39)	721 (38.74)	
Female	4800 (65.25)	3660 (66.61)	1140 (61.26)	
Race, *n* (%)				<0.001
White	5334 (72.51)	4124 (75.05)	1210 (65.02)	
Black	444 (6.04)	294 (5.35)	150 (8.06)	
Others	1578 (21.45)	1077 (19.60)	501 (26.92)	
Weight (kg)	91.00 ± 22.53	91.29 ± 21.64	90.13 ± 24.95	0.074
Comorbidity				
Aki (%)	5953 (80.93)	4262 (77.56)	1691 (90.87)	<0.001
Myocardial infarct (%)	1453 (19.75)	1125 (20.47)	328 (17.62)	0.008
Congestive heart failure (%)	1830 (24.88)	1309 (23.82)	521 (28.00)	<0.001
Cerebrovascular disease (%)	936 (12.72)	569 (10.35)	367 (19.72)	<0.001
Chronic pulmonary disease (%)	1692 (23.00)	1215 (22.11)	477 (25.63)	0.002
Diabetes (%)	2229 (30.30)	1644 (29.92)	585 (31.43)	0.219
Renal disease (%)	1133 (15.40)	785 (14.29)	348 (18.70)	<0.001
Liver disease (%)	280 (3.81)	131 (2.38)	149 (8.01)	<0.001
Malignant cancer (%)	527 (7.16)	346 (6.30)	181 (9.73)	<0.001
Laboratory tests				
Glucose, mg/dL	149.05 ± 60.62	145.17 ± 53.14	160.50 ± 77.55	<0.001
Lactate, mmol/L	2.52 ± 1.75	2.40 ± 1.56	2.88 ± 2.19	<0.001
BUN, mg/dL	21.39 ± 16.51	20.14 ± 14.51	25.08 ± 20.93	<0.001
Creatinine, mg/dL	1.14 ± 1.04	1.09 ± 1.01	1.28 ± 1.10	<0.001
Bicarbonate, mmol/L	22.43 ± 3.85	22.82 ± 3.45	21.25 ± 4.64	<0.001
Chloride, mmol/L	107.38 ± 6.34	108.10 ± 5.61	105.26 ± 7.75	<0.001
Calcium, mg/dL	8.22 ± 0.82	8.22 ± 0.74	8.23 ± 1.02	0.784
Potassium, mmol/L	4.25 ± 0.67	4.25 ± 0.62	4.24 ± 0.80	0.833
WBC, K/μL	12.10 (8.80, 16.10)	12.00 (8.80, 15.80)	12.40 (8.60, 17.10)	0.014
Platelet, K/μL	156.00 (119.00, 209.00)	155.00 (120.00, 204.00)	160.00 (113.00, 227.00)	0.157
RBC, m/μL	3.27 (2.82, 3.77)	3.25 (2.82, 3.71)	3.36 (2.81, 3.93)	<0.001
Urine output, mL/24 h	1770.00 (1217.00, 2500.00)	1818.00 (1295.00, 2515.00)	1575.00 (970.00, 2435.00)	<0.001
Sodium first, mmol/L	136.85 ± 4.89	137.08 ± 4.16	136.14 ± 6.54	<0.001
Sodium min, mmol/L	134.98 ± 4.91	135.10 ± 3.88	134.64 ± 7.12	0.008
Sodium max, mmol/L	137.48 ± 11.63	137.27 ± 12.88	138.10 ± 6.66	<0.001
Sodium average, mmol/L	136.22 ± 6.94	136.18 ± 7.19	136.35 ± 6.13	0.318
Disease severity score				
Sofa score	3.00 (2.00, 5.00)	3.00 (2.00, 5.00)	4.00 (3.00, 6.00)	<0.001
SAPS II score	38.00 (31.00, 47.00)	37.00 (31.00, 45.00)	42.00 (34.00, 52.00)	<0.001
Charlson comorbidity index	4.00 (3.00, 6.00)	4.00 (2.00, 6.00)	5.00 (3.00, 7.00)	<0.001
Intervention				
Midazolam exposure (%)	1150 (15.63)	644 (11.72)	506 (27.19)	<0.001
Mechanical ventilation (%)	7103 (96.56)	5321 (96.83)	1782 (95.75)	0.027
Outcomes				
Hospital LOS	7.99 (5.29, 13.77)	6.98 (5.10, 10.82)	14.18 (8.16, 23.76)	<0.001
ICU LOS	2.52 (1.37, 5.39)	2.15 (1.30, 3.60)	6.47 (3.44, 12.05)	<0.001
Hospital Mortality (%)	620 (8.43)	347 (6.31)	273 (14.67)	<0.001
ICU Mortality (%)	467 (6.35)	281 (5.11)	186 (9.99)	<0.001
28-day Mortality (%)	686 (9.33)	386 (7.02)	300 (16.12)	<0.001

Continuous variables are presented as mean (standard deviation) or median (interquartile range); categorical variables are shown as count (percentage). ICU, intensive care unit; LOS, length of stay; SAPS II, Simplified Acute Physiology Score II; SOFA, Sequential Organ Failure Assessment; BUN, blood urea nitrogen; WBC, white blood cell count; RBC, red blood cell count.

**Table 2:** Clinical outcomes of patients with different sodium levels.

Variables	Total	Hyponatremia	Normal	Hypernatremia	*p*
Number	7356	1577	5623	156	
Delirium (%)	1861 (25.30)	582 (36.91)	1195 (21.25)	84 (53.85)	<0.001
Hospital LOS	7.99 (5.29, 13.77)	11.11 (6.70, 19.67)	7.29 (5.18, 12.06)	10.25 (4.70, 19.06)	<0.001
ICU LOS	2.52 (1.37, 5.39)	3.55 (1.99, 7.59)	2.31 (1.32, 4.89)	4.19 (2.47, 8.62)	<0.001
Hospital Mortality (%)	620 (8.43)	236 (14.97)	324 (5.76)	60 (38.46)	<0.001
ICU Mortality (%)	467 (6.35)	163 (10.34)	256 (4.55)	48 (30.77)	<0.001
28-day Mortality (%)	686 (9.33)	258 (16.36)	362 (6.44)	66 (42.31)	<0.001

Hyponatremia: sodium < 135 mmol/L, Normal: 135 mmol/L ≤ Sodium ≤ 145 mmol/L, Hypernatremia: Sodium > 145 mmol/L. Continuous variables are presented as mean (standard deviation) or median (interquartile range), while categorical variables are shown as count (percentage). ICU, intensive care unit; LOS, length of stay.

**Table 3:** Logistic regression analysis of sodium levels and delirium development in sepsis patients.

Variables	Model 1		Model 2		Model 3		Model 4	
	OR (95% CI)	*p*	OR (95% CI)	*p*	OR (95% CI)	*p*	OR (95% CI)	*p*
Hyponatremia	Reference							
Normal	0.46 (0.41~0.52)	<0.001	0.52 (0.46~0.58)	<0.001	0.77 (0.67~0.89)	<0.001	0.79 (0.68~0.92)	0.002
Hypernatremia	1.99 (1.43~2.78)	<0.001	1.77 (1.26~2.49)	0.001	3.45 (2.35~5.06)	<0.001	3.37 (2.28~5.00)	<0.001

OR: Odds Ratio, CI: Confidence Interval. Hyponatremia: sodium < 135 mmol/L, Normal: 135 mmol/L ≤ Sodium ≤ 145 mmol/L, Hypernatremia: Sodium > 145 mmol/L. Model 1: Crude. Model 2: Adjusted for gender, race, age, weight, and comorbidities including AKI, myocardial infarction, congestive heart failure, cerebrovascular disease, chronic pulmonary disease, diabetes, renal disease, liver disease, and malignant cancer. Model 3: Built upon Model 2, adjusting for additional laboratory parameters such as chloride levels, white blood cells (WBC), calcium, potassium, platelets, and red blood cells (RBC). Model 4: Built upon Model 3, with further adjustments for midazolam use in ICU, ventilator use, and SOFA score.

**Table 4:** Baseline characteristics and clinical outcomes in PSM cohorts.

Variables	Overall	Hyponatremia	Hypernatremia	*p*
Number	120	60	60	
Age, years	59.18 ± 18.94	58.97 ± 19.62	59.40 ± 18.39	0.901
Gender, *n* (%)				0.583
Male	63 (52.5)	30 (50.00)	33 (55.00)	
Female	57 (47.5)	30 (50.00)	27 (45.00)	
Race, *n* (%)				0.765
White	62 (51.67)	33 (55.00)	29 (48.33)	
Black	17 (14.17)	8 (13.33)	9 (15.00)	
Others	41 (34.17)	19 (31.67)	22 (36.67)	
Weight (kg)	79.10 (67.22, 97.62)	79.80 (66.97, 94.97)	78.15 (67.38, 98.35)	0.964
Comorbidity				
Aki Flag (%)	106 (88.33)	54 (90.00)	52 (86.67)	0.570
Myocardial infarct (%)	14 (11.67)	6 (10.00)	8 (13.33)	0.570
Congestive heart failure (%)	31 (25.83)	16 (26.67)	15 (25.00)	0.835
Cerebrovascular disease (%)	38 (31.67)	20 (33.33)	18 (30.00)	0.695
Chronic pulmonary disease (%)	26 (21.67)	13 (21.67)	13 (21.67)	1.000
Diabetes (%)	26 (21.67)	11 (18.33)	15 (25.00)	0.375
Renal disease (%)	20 (16.67)	11 (18.33)	9 (15.00)	0.624
Liver disease (%)	6 (5)	2 (3.33)	4 (6.67)	0.675
Malignant cancer (%)	12 (10)	6 (10.00)	6 (10.00)	1.000
Laboratory tests				
Glucose, mg/dL	185.65 ± 109.16	179.78 ± 87.56	191.52 ± 127.65	0.558
Lactate, mmol/L	3.53 ± 2.94	3.57 ± 2.62	3.50 ± 3.25	0.894
BUN, mg/dL	29.81 ± 30.77	29.67 ± 33.46	29.95 ± 28.11	0.960
Creatinine, mg/dL	1.34 ± 1.13	1.38 ± 1.23	1.31 ± 1.03	0.743
Bicarbonate, mmol/L	20.93 ± 4.68	20.88 ± 3.83	20.97 ± 5.43	0.923
Chloride, mmol/L	108.45 ± 6.62	107.88 ± 6.42	109.02 ± 6.82	0.350
Calcium, mg/dL	8.40 ± 1.12	8.41 ± 1.29	8.39 ± 0.94	0.936
Potassium, mmol/L	4.00 ± 0.81	4.00 ± 0.79	3.99 ± 0.84	0.947
WBC, K/μL	12.40 (7.97, 16.97)	12.35 (7.70, 15.98)	12.60 (8.52, 17.73)	0.571
Platelet, K/μL	166.50 (117.00, 224.75)	164.50 (109.50, 224.00)	170.50 (126.75, 232.25)	0.885
RBC, m/μL	3.54 (2.94, 4.34)	3.59 (2.99, 4.20)	3.54 (2.92, 4.42)	0.763
Urine output, mL/24 h	2242.50 (1240.00, 3285.00)	2315.00 (1300.00, 2996.25)	2172.00 (1231.25, 3407.50)	0.618
Disease severity score				
Sofa score	3.00 (2.00, 5.00)	3.00 (2.00, 4.25)	4.00 (3.00, 5.00)	0.245
SAPA II score	41.00 (33.75, 52.00)	43.00 (34.75, 55.25)	40.50 (33.00, 48.25)	0.583
Charlson comorbidity index	4.00 (2.00, 6.00)	4.00 (2.00, 6.00)	4.00 (2.00, 7.00)	0.682
Intervention				
Midazolam exposure (%)	30 (25)	17 (28.33)	13 (21.67)	0.399
Mechanical ventilation (%)	108 (90)	53 (88.33)	55 (91.67)	0.543
Outcomes				
Delirium (%)	55 (45.83)	22 (36.67)	33 (55.00)	0.044
Hospital LOS	13.95 (6.55, 23.50)	15.46 (6.85, 23.61)	11.91 (5.28, 23.37)	0.261
ICU LOS	5.54 (2.44, 11.27)	4.77 (2.46, 16.53)	5.96 (2.37, 9.55)	0.696
Hospital Mortality (%)	33 (27.50)	12 (20.00)	21 (35.00)	0.066
ICU Mortality (%)	28 (23.33)	10 (16.67)	18 (30.00)	0.084
28-day Mortality (%)	33 (27.50)	12 (20.00)	21 (35.00)	0.066

## Data Availability

The data used and analyzed in this study are available from the corresponding author on request.

## Supplementary Materials

The following supporting information can be downloaded at: https://www.mdpi.com/article/10.3390/biomedicines14020410/s1, Additional File S1; Additional File S2; Table S1: Logistic regression analysis of different serum sodium stratification methods and their association with delirium in septic patients; Table S2: Logistic regression analysis of different serum sodium stratification methods and delirium risk in mechanically ventilated septic patients.

## Abbreviations

The following abbreviations are used in this manuscript:

SSD	Subsyndromal delirium
RCS	Restricted cubic spline
