# Supplementary material for: Association of Serum Sodium Levels and Delirium in Patients with Sepsis: A Retrospective Study
Source: Biomedicines. 2026 Feb 11;14(2):410. doi: 10.3390/biomedicines14020410 (PMC12937704; doi:10.3390/biomedicines14020410)
Supplement: Supplementary file 1 [file biomedicines-14-00410-s001.zip › Additional file S1.pdf]

CITI PROGRAM

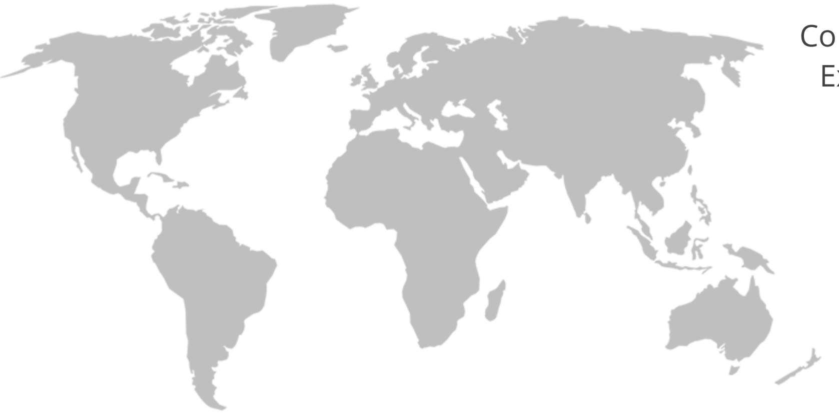

Completion Date 24-Apr-2025
Expiration Date 24-Apr-2028
Record ID 69192968

This is to certify that:

Meiying Wang

Not valid for renewal of certification through CME.

Has completed the following CITI Program course:

Human Research
(Curriculum Group)
Data or Specimens Only Research
(Course Learner Group)
2 - Refresher Course
(Stage)

Under requirements set by:

Massachusetts Institute of Technology Affiliates

CITI
Collaborative Institutional Training Initiative
101 NE 3rd Avenue, Suite 320
Fort Lauderdale, FL 33301 US
www.citiprogram.org

Generated on 04-May-2025. Verify at www.citiprogram.org/verify/?wb3b8e2b0-c36d-4d5a-be00-0bd446695f59-69192968
