# Supplementary material for: Association of Serum Sodium Levels and Delirium in Patients with Sepsis: A Retrospective Study
Source: Biomedicines. 2026 Feb 11;14(2):410. doi: 10.3390/biomedicines14020410 (PMC12937704; doi:10.3390/biomedicines14020410)
Supplement: Supplementary file 1 [file biomedicines-14-00410-s001.zip › Additional file S2.pdf]

Table S1 Logistic Regression Analysis of Different Serum Sodium Stratification Methods and Their Association with Delirium in Septic Patients

Variables	Model1		Model2		Model3		Model4	
	OR (95%CI)	*P*	OR (95%CI)	*P*	OR (95%CI)	*P*	OR (95%CI)	*P*
Q1	Reference							
Q2	0.52 (0.44 ~ 0.62)	<.001	0.59 (0.50 ~ 0.70)	<.001	0.77 (0.65 ~ 0.93)	0.006	0.78 (0.65 ~ 0.94)	0.008
Q3	0.40 (0.34 ~ 0.47)	<.001	0.48 (0.41 ~ 0.56)	<.001	0.67 (0.56 ~ 0.80)	<.001	0.70 (0.58 ~ 0.84)	<.001
Q4	0.54 (0.47 ~ 0.62)	<.001	0.59 (0.51 ~ 0.69)	<.001	0.88 (0.74 ~ 1.05)	0.150	0.90 (0.76 ~ 1.07)	0.247
Sodium ≤ 138 mmol/L	1.12 (1.09 ~ 1.15)	<.001	1.09 (1.07 ~ 1.12)	<.001	1.12 (1.09 ~ 1.15)	<.001	1.08 (1.06 ~ 1.11)	<.001
Sodium >138 mmol/L	0.91 (0.89 ~ 0.92)	<.001	0.92 (0.90 ~ 0.93)	<.001	0.97 (0.95 ~ 0.99)	0.020	0.97 (0.95 ~ 0.99)	0.041

OR: Odds Ratio, CI: Confidence Interval,Q1:Sodium≤135 mmol/L, Q2: 135 mmol/L < Sodium ≤ 137 mmol/L, Q3:137 mmol/L < Sodium ≤ 139 mmol/L, Q4: Sodium ≥ 139 mmol/L. Model1: Crude. Model2: Adjust: gender, race, age, weight, and comorbidities such as AKI, myocardial infarct, congestive heart failure, cerebrovascular disease, chronic pulmonary disease, diabetes, renal disease, liver disease, malignant cancer. Model3: Built upon Model 2 by adjusting for additional laboratory parameters, such as chloride levels, white blood cells (WBC), calcium, potassium, platelets, red blood cells (RBC). Model4: Built upon Model 3 by further adjusting for midazolam icu used, ventilator flag, SOFA score

Table S2 Table X. Logistic Regression Analysis of Different Serum Sodium Stratification Methods and Delirium Risk in Mechanically Ventilated Septic Patients

Variables	Model1		Model2		Model3		Model4	
	OR (95%CI)	*P*	OR (95%CI)	*P*	OR (95%CI)	*P*	OR (95%CI)	*P*
Q1	Reference							
Q2	0.49 (0.41 ~ 0.57)	<.001	0.55 (0.46 ~ 0.65)	<.001	0.74 (0.62 ~ 0.90)	0.002	0.75 (0.62 ~ 0.91)	0.003
Q3	0.38 (0.33 ~ 0.45)	<.001	0.46 (0.39 ~ 0.54)	<.001	0.67 (0.56 ~ 0.80)	<.001	0.70 (0.59 ~ 0.85)	<.001
Q4	0.50 (0.44 ~ 0.58)	<.001	0.56 (0.48 ~ 0.65)	<.001	0.88 (0.74 ~ 1.05)	0.153	0.90 (0.75 ~ 1.08)	0.267
Sodium ≤ 138 mmol/L	0.89 (0.87 ~ 0.91)	<.001	0.90 (0.89 ~ 0.92)	<.001	0.96 (0.93 ~ 0.99)	0.002	0.96 (0.94 ~ 0.99)	0.008
Sodium >138 mmol/L	1.13 (1.10 ~ 1.16)	<.001	1.10 (1.07 ~ 1.13)	<.001	1.13 (1.10 ~ 1.17)	<.001	1.12 (1.09 ~ 1.16)	<.001

OR: Odds Ratio, CI: Confidence Interval,Q1:Sodium≤135 mmol/L, Q2: 135 mmol/L < Sodium ≤ 137 mmol/L, Q3:137 mmol/L < Sodium ≤ 139 mmol/L, Q4: Sodium ≥ 139 mmol/L. Model1: Crude. Model2: Adjust: gender, race, age, weight, and comorbidities such as AKI, myocardial infarct, congestive heart failure, cerebrovascular disease, chronic pulmonary disease, diabetes, renal disease, liver disease, malignant cancer. Model3: Built upon Model 2 by adjusting for additional laboratory parameters, such as chloride levels, white blood cells (WBC), calcium, potassium, platelets, red blood cells (RBC). Model4: Built upon Model 3 by further adjusting for midazolam icu used, ventilator flag, SOFA sco
